# Research Progress of Natural Polysaccharide-Based Hydrogels in Skin Tissue Regeneration

**DOI:** 10.3390/gels12010021

**Published:** 2025-12-25

**Authors:** Xushuang Jia, Dongmei Fan, Zhuoya Yang, Junjie Chang, Qi Wang, Xiaohan Cui, Da Liu, Ning Cui, Ye Jin

**Affiliations:** 1 School of Pharmacy, Changchun University of Chinese Medicine, Changchun 130117, China; 23203070205@stu.ccucm.edu.cn (X.J.); 15804440604@163.com (D.F.); 15584356629@163.com (Z.Y.); 17549647176@163.com (J.C.); 19819514897@163.com (Q.W.); jia07300909@163.com (X.C.); liuda@ccucm.edu.cn (D.L.); 2 Public Experimental Center, Changchun University of Chinese Medicine, Changchun 130117, China; 3 Northeast Asian Institute of Traditional Chinese Medicine, Changchun University of Chinese Medicine, Changchun 130117, China

**Keywords:** natural polysaccharide hydrogels, skin tissue regeneration, wound healing, anti-inflammatory, angiogenesis, oxidative stress, extracellular matrix remodeling

## Abstract

Disorders of skin wound healing and the repair of full-thickness skin defects remain significant clinical challenges. Natural polysaccharide-based hydrogels, with their excellent biocompatibility, tunable degradability, and multifunctional properties (e.g., antibacterial, antioxidant, and pro-angiogenic), have emerged as key materials for designing wound dressings and skin tissue engineering scaffolds. This review systematically summarizes recent advances in polysaccharide hydrogels—including chitosan, hyaluronic acid, and alginate—focusing on material types, crosslinking strategies, and functional modifications, with particular emphasis on their dual applications in wound healing (acute and chronic wounds) and skin tissue engineering. In wound healing, these hydrogels regulate the microenvironment through multiple mechanisms, including anti-inflammatory, antioxidant, pro-angiogenic, and immunomodulatory effects. In skin tissue engineering, their three-dimensional porous structures mimic the extracellular matrix, supporting cell adhesion, proliferation, and tissue regeneration. Finally, we discuss the challenges and future prospects for the clinical translation and commercialization of natural polysaccharide hydrogels.

## 1. Introduction

**Figure 1:**
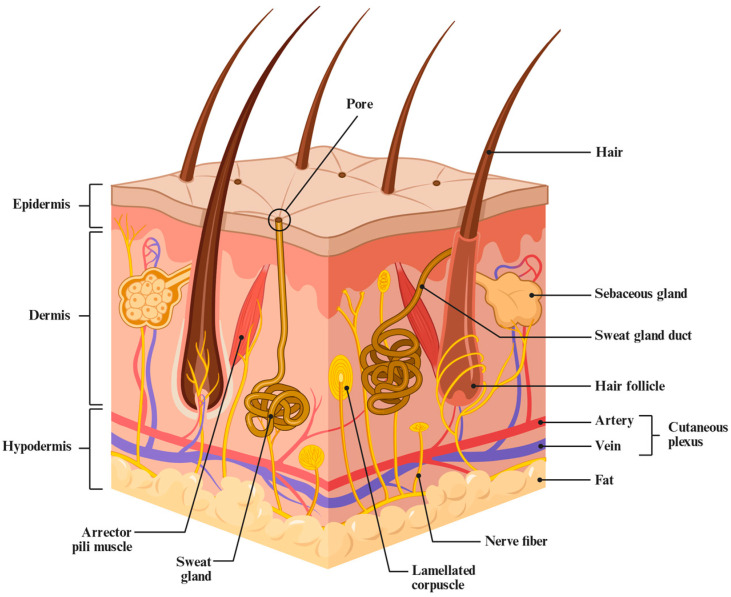
Schematic Illustration of Human Skin Anatomy and Layers Relevant to Wound Healing.

The skin, as the largest organ of the human body, plays an indispensable role in defending against physical, chemical, and biological insults, maintaining water and electrolyte balance, regulating body temperature, sensing environmental stimuli, and participating in immune defense (Figure 1) [1,2]. When the skin is structurally damaged due to trauma, burns, surgical procedures, or chronic diseases such as diabetes, its barrier function is compromised, making it highly susceptible to imbalanced inflammatory responses, exudate loss, bacterial infection, and tissue necrosis, which may lead to chronic non-healing wounds or even systemic complications.

Therefore, developing effective, safe, and long-term applicable wound repair strategies is of great clinical significance for restoring the structural integrity and functional homeostasis of the skin. With the advent of the moist healing theory and regenerative medicine concepts, modern wound care materials are no longer limited to passive physical coverage or isolation, but are expected to actively modulate the wound microenvironment and promote tissue regeneration [3,4]. An ideal wound dressing or skin tissue engineering scaffold should possess good biocompatibility, appropriate mechanical properties, effective exudate management, and the ability to regulate infection and inflammation. Moreover, in the repair of complex or chronic wounds, the material should support cell adhesion, migration, and proliferation, and provide stage-specific regulation at different healing phases.

In this context, hydrogels, with their high water content, three-dimensional network structures, and ability to mimic the natural extracellular matrix (ECM), are considered highly promising materials for wound repair and skin tissue engineering [5,6]. Particularly, injectable, self-healing, or bioactive-loaded hydrogel systems offer new technological avenues for minimally invasive treatment and precise tissue repair.

Among various hydrogel systems, natural polysaccharide-based hydrogels have attracted sustained attention due to their abundant sources, excellent biocompatibility, good biodegradability, and low immunogenicity [7]. Common natural polysaccharides include chitosan [8], hyaluronic acid (HA) [9], sodium alginate [10], cellulose and its derivatives [4], dextran, and others [11]. These polysaccharide molecules are rich in hydroxyl, amino, and carboxyl functional groups, allowing their physicochemical properties and biological functions to be tailored via oxidation, grafting, or ionic crosslinking. Different polysaccharides exhibit unique biological advantages in skin repair. For example, chitosan, due to its cationic nature, can disrupt bacterial cell membranes through electrostatic interactions, exhibiting inhibitory rates over 95% and 90% against *Staphylococcus aureus* and *Escherichia coli*, respectively, and shows over 85% antioxidant activity in DPPH radical scavenging assays, demonstrating its ‘antibacterial-antioxidant’ synergistic effect [12]. Hyaluronic acid can interact with cell surface receptors such as CD44 to regulate cell migration and angiogenesis [13,14]. In addition, functional polysaccharides derived from medicinal and edible or traditional Chinese medicine sources, such as fucoidan [15], astragalus polysaccharide [16], and Ganoderma lucidum polysaccharide [17], possess innate immunomodulatory, antioxidant, and pro-repair activities, providing natural advantages for integrating material design with therapeutic functionality. The application value of natural polysaccharide-based hydrogels extends beyond precise intervention in acute and chronic wound healing, to include functional upgrades of wound dressings (e.g., injectable, self-healing, and smart-responsive dressings) and biomimetic construction of skin tissue engineering scaffolds (e.g., mimicking ECM and supporting vascularized regeneration). Its integrated “material design–mechanistic modulation–application adaptation” feature enables simultaneous improvement of the local wound microenvironment, fulfillment of clinical dressing requirements, and support for tissue engineering regeneration, making it a key bridge between fundamental research and clinical translation.

To further enhance the adaptability and functionality of natural polysaccharide hydrogels in complex wound repair, researchers have developed various crosslinking and functionalization strategies, including physical crosslinking (e.g., hydrogen bonding, hydrophobic interactions, and ionic bonds) and chemical crosslinking (e.g., dynamic Schiff base covalent bonds, boronate ester bonds, and free-radical polymerization). In particular, the incorporation of dynamic covalent bonds endows hydrogels with self-healing ability, injectability, and responsiveness to pH, redox conditions, or metabolite-related cues, offering distinct advantages in complex microenvironments such as diabetic and infected wounds [18,19,20,21].

Meanwhile, the three-dimensional porous structures formed by natural polysaccharide hydrogels not only serve as physical scaffolds to support cell adhesion, migration, and proliferation but can also be integrated with nanotechnology, sprayable systems, and microneedle arrays to achieve localized and controlled delivery of antibacterial agents, growth factors, stem cell-derived exosomes, nucleic acids, or other bioactive molecules, thereby enhancing therapeutic efficacy while reducing systemic side effects [22,23,24,25,26,27,28].

At the biological mechanism level, natural polysaccharide hydrogels synergistically regulate wound repair through multiple targets and pathways, including immunomodulation, antioxidant activity, antimicrobial effects, promotion of angiogenesis, and ECM remodeling. For example, they can promote the polarization of macrophages from pro-inflammatory M1 to reparative M2 phenotype via regulation of the NF-κB signaling pathway [29]. They can also alleviate oxidative stress by activating the Nrf2/HO-1 pathway [30]; and inhibit bacterial growth and biofilm formation [31,32]. Moreover, they can promote angiogenesis and tissue regeneration by upregulating VEGF and stabilizing HIF-1α [33,34,35]. In combination with controlled-release designs, hydrogels can achieve sustained delivery of bioactive substances, providing precise intervention at different stages of wound healing [36,37].

In recent years, natural polysaccharide-based composite hydrogels have evolved from single-material systems into multifunctional, programmable, and responsive integrated therapeutic platforms, showing remarkable progress in models of diabetic wounds, infected wounds, and full-thickness skin defects [36,37,38,39,40]. However, challenges remain in terms of long-term stability, adaptability across diverse clinical scenarios, and scalable translation [38,39,40,41,42]. Based on this, the present review focuses on three core aspects: intervention in wound healing mechanisms, functional optimization of wound dressings, and construction of skin tissue engineering scaffolds. It systematically summarizes the material types, crosslinking and functionalization strategies, mechanisms of action, and the latest applications of natural polysaccharide-based hydrogels in acute wounds, chronic wounds, functional dressings, and tissue engineering, and further discusses their translational potential and future development directions.

## 2. Pathogenesis of Impaired Skin Wound Healing and Therapeutic Targets

**Figure 2:**
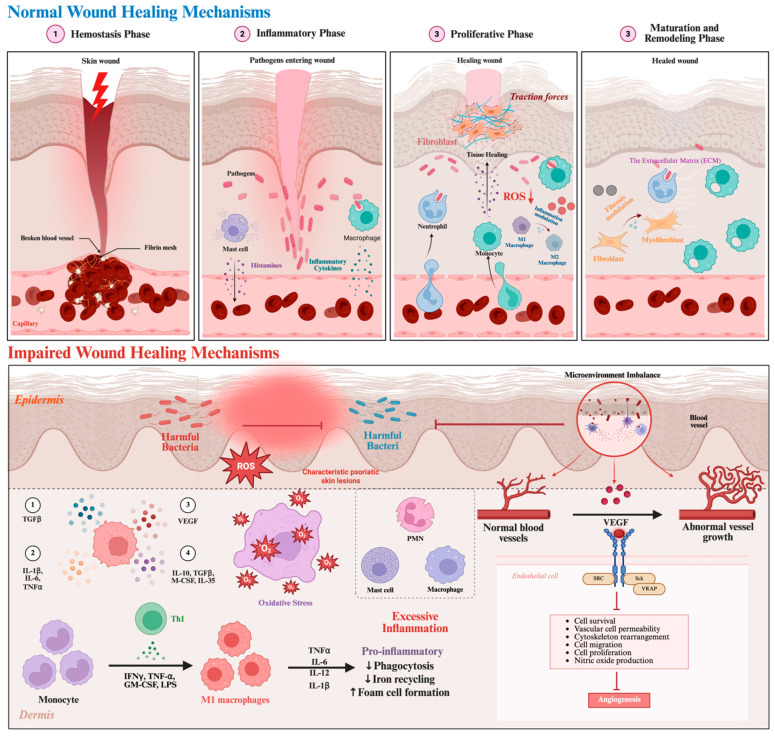
Physiological and pathological mechanisms of skin wound healing. The schematic illustrates the four sequential phases of normal wound healing—hemostasis, inflammation, proliferation, and remodeling—and highlights common pathological features leading to impaired healing, such as persistent inflammation, oxidative stress, infection, and extracellular matrix disruption.

Skin wound healing is a highly coordinated and dynamic biological process involving multiple cell types, signaling pathways, and interactions with the extracellular matrix (ECM), aimed at rapidly restoring the skin barrier function and reestablishing tissue integrity. This process is typically divided into four overlapping and tightly regulated phases: hemostasis, inflammation, proliferation, and remodeling. Each phase is dominated by specific cell populations and progresses in an orderly manner through complex intercellular signaling networks and microenvironmental regulation [43] (Figure 2).

### 2.1. Normal Cascade Reactions of Wound Healing

After injury, the body immediately initiates the hemostatic response. Vascular smooth muscles undergo reflexive contraction to reduce bleeding [44]. Exposed subcutaneous matrix components, such as collagen, activate platelets, inducing morphological changes and mediating adhesion and aggregation via integrins (e.g., αIIbβ3) and glycoprotein complexes (e.g., GPIb-IX-V), forming a platelet plug to achieve primary hemostasis [45,46]. Simultaneously, the coagulation cascade is activated, and the generated thrombin converts fibrinogen into fibrin, forming a stable clot to complete secondary hemostasis.

In addition to physical blockage, activated platelets release a variety of bioactive molecules, such as platelet-derived growth factor (PDGF), transforming growth factor-β (TGF-β), and epidermal growth factor (EGF), which provide signaling cues for subsequent recruitment of inflammatory cells and tissue repair [46]. Following the completion of hemostasis, the inflammatory response is rapidly initiated. Neutrophils migrate first to the wound under the guidance of chemokines (e.g., ELR+ CXC family) and damage-associated molecular patterns (DAMPs), where they phagocytose pathogens, release granule contents such as myeloperoxidase and elastase, and form neutrophil extracellular traps (NETs) to clear necrotic tissue and microbes [47,48]. Subsequently, monocytes are recruited and differentiate into macrophages. Early M1 macrophages secrete pro-inflammatory factors (e.g., TNF-α, IL-1β) to enhance immune defense and promote the resolution of inflammation by engulfing apoptotic neutrophils (efferocytosis) [49]. As inflammation gradually subsides, macrophages further polarize into the M2 phenotype, releasing repair factors such as vascular endothelial growth factor (VEGF) and PDGF to promote angiogenesis and tissue regeneration, laying the foundation for the proliferative phase [50]. The proliferative phase is the core period of wound repair, mainly including granulation tissue formation, angiogenesis, and re-epithelialization. Stimulated by growth factors secreted by platelets and macrophages, fibroblasts are activated and migrate into the provisional fibrin-based matrix, synthesizing and secreting ECM components such as collagen III and fibronectin to form granulation tissue [51]. Meanwhile, hypoxic conditions combined with VEGF signaling promote endothelial cell sprouting, forming a capillary network to supply oxygen and nutrients to the repairing tissue [52]. Some fibroblasts differentiate into myofibroblasts under TGF-β signaling and mechanical tension, expressing α-smooth muscle actin (α-SMA) and contributing to wound contraction [53].

In addition, keratinocytes from the wound edges and the hair follicle bulge region are activated, detach from the basement membrane, and migrate to cover the wound, expressing keratins K6, K16, and K17, and upregulating integrins (α5β1, αvβ6) to promote adhesion and migration [54]. As these cells migrate and proliferate, the epidermis eventually restores its stratified structure, forming a complete skin barrier [55]. Although re-epithelialization is achieved, connective tissue continues remodeling over the following months to years. Early-deposited collagen III is gradually replaced by mechanically stronger collagen I, and collagen fibers are realigned and crosslinked under mechanical tension to enhance tissue mechanical stability. Matrix metalloproteinases (MMPs) and their tissue inhibitors (TIMPs) play key roles in maintaining ECM dynamic balance, ensuring gradual optimization of tissue structure [56]. Upon completion of repair, excess blood vessels are pruned, and myofibroblasts are cleared via apoptosis, ultimately forming a mature scar characterized by low cell density and dense structure [57]. Overall, normal wound healing is a continuous process of multi-cell cooperation and precisely regulated signaling networks. Any abnormality at a given phase, such as prolonged inflammation, insufficient cell migration, or imbalance in matrix remodeling, may result in impaired wound healing, leading to chronic wounds or pathological scarring [43].

### 2.2. Pathological Mechanisms of Chronic Wounds

Wound healing impairment results from abnormalities in multiple factors and regulatory pathways, involving a combined effect of local microenvironment imbalance and systemic pathological factors. One of the core mechanisms is excessive inflammation. In chronic wounds, pro-inflammatory factors remain persistently elevated, and macrophage phenotype switching is impaired, preventing the transition from a pro-inflammatory to a reparative state, which prolongs the inflammatory phase and disrupts the tissue repair microenvironment [58]. Limited angiogenesis directly affects local oxygen and nutrient supply. Under pathological conditions such as diabetes, aging, and vascular diseases, VEGF and related signaling pathways are impaired, endothelial cell function is compromised, and neovascular formation is insufficient, exacerbating local hypoxia and ischemia [59]. Impaired cell migration and proliferation are also critical. Inflammatory factors, high-glucose environments, and oxidative stress inhibit keratinocyte migration and fibroblast proliferation, leading to delayed re-epithelialization and insufficient granulation tissue formation [60]. Oxidative stress and microbial infection form a vicious cycle. Reactive oxygen species damage cell membranes and mitochondrial function, while pathogens such as *Staphylococcus aureus* and *Pseudomonas aeruginosa* form biofilms that resist immune clearance and antibiotic action through extracellular polysaccharide matrices. They also release proteases and other virulence factors to degrade repair-related molecules, continuously inducing chronic inflammation and further inhibiting cell migration and proliferation [61]. Chronic systemic diseases and metabolic abnormalities exacerbate healing impairment through multiple pathways. Accumulation of advanced glycation end products (AGEs) in diabetes can activate the RAGE receptor, amplifying oxidative stress and inflammatory cascades while inhibiting fibroblast function and collagen deposition. Neuropathy and sensory loss increase the risk of repeated injury while weakening neuro-immune-vascular coordinated repair mechanisms. Malnutrition directly affects collagen synthesis and immune cell activity [62]. At the molecular level, NF-κB remains continuously activated in chronic wounds, inducing excessive release of matrix metalloproteinases (MMPs) by neutrophils, degrading ECM and growth factors while suppressing anti-inflammatory factor expression, forming a vicious “inflammation-damage” cycle. Angiogenesis-related signaling pathways (VEGF, HIF-1α, PI3K/Akt, etc.) are suppressed, hindering endothelial cell migration and lumen formation. Cell migration and proliferation signals (Rac1/CDC42, TGF-β/Smad, Cyclin/CDK complexes) are dysregulated, leading to impaired keratinocyte and fibroblast function, reduced granulation tissue formation, and compromised wound contraction [58,62].

Furthermore, wound microbiome imbalance plays a key role in chronicity. Microbial diversity decreases, pathogenic bacteria dominate and form biofilms, regulating virulence factor expression through quorum sensing, inhibiting host immune responses, and degrading repair-related molecules, further delaying healing. Interactions between microbiota and host are complex; high-glucose environments promote pathogen colonization, while bacterial metabolites can activate host TLR/NF-κB signaling, amplifying local inflammation [63]. In summary, chronic wound healing impairment arises from the interplay of excessive inflammation, limited angiogenesis, impaired cell migration and proliferation, oxidative stress, microbiome imbalance, and chronic systemic pathological factors. These mechanisms collectively shift the wound from acute repair toward chronic stagnation, providing a theoretical basis for developing multi-targeted intervention strategies [64].

### 2.3. Key Biological Targets in Skin Regeneration

Wound repair is a complex process involving inflammation regulation, angiogenesis, oxidative balance, and tissue reconstruction. Its key biological targets can be summarized into four functional modules, which synergistically drive the transition of the wound from the inflammatory phase to the proliferative and remodeling phases through signaling networks [65]. In terms of anti-inflammation and immune regulation, TNF-α, IL-1β, and the NF-κB signaling pathway are core targets. In chronic or diabetic wounds, these pro-inflammatory factors are abnormally overexpressed, persistently activating NF-κB, inhibiting fibroblast proliferation and angiogenesis, and prolonging the inflammatory phase [66]. The balance of M1/M2 macrophage polarization is also crucial: M1 macrophages secrete pro-inflammatory factors to clear necrotic tissue, whereas M2 macrophages release TGF-β and VEGF to promote tissue repair [67]. Delayed M1-to-M2 polarization in chronic wounds is a major cause of impaired healing; modulation of transcription factors such as PPARγ can restore macrophage function [68,69].

Regarding angiogenesis, VEGF, bFGF, and HIF-1α are the main targets. VEGF binds VEGFR2 to activate the PI3K/Akt pathway, promoting endothelial cell migration and lumen formation; its expression is impaired under high oxidative stress and diabetic conditions [5,6]. HIF-1α responds to hypoxic signals, upregulating VEGF and eNOS expression. Stabilization of HIF-1α or pharmacological interventions (e.g., exosomes or deferoxamine) can improve neovascularization in ischemic wounds [70]. bFGF synergizes with VEGF to enhance fibroblast activity and support newly formed vessels [71].

For oxidative stress protection, excessive ROS damages the wound microenvironment through lipid peroxidation and DNA damage [72]. The Nrf2/HO-1 signaling pathway serves as a major endogenous defense mechanism: under oxidative stress, Nrf2 dissociates from Keap1, translocates into the nucleus, and upregulates the expression of antioxidant enzymes such as HO-1 and SOD [73]. Exogenous interventions, such as N-acetylcysteine or copper-based nanozymes, can scavenge ROS, while Nrf2 activators (e.g., bardoxolone methyl) can reduce oxidative damage and promote repair [74].

In terms of ECM reconstruction and epithelial regeneration, collagen deposition, MMP regulation, and keratinocyte migration are key. Ordered deposition of collagen I/III forms the basis of repaired tissue. In diabetic wounds, excessive MMP-9 activation leads to collagen degradation imbalance, which can be mitigated by selective MMP-9 inhibitors (e.g., ND-336) to preserve ECM integrity [75]. Keratinocyte migration depends on EGF and integrin signaling and is regulated by the microenvironment guided by pericytes via the VEGF/Dll4/Notch pathway; dysfunction leads to delayed epithelial regeneration. In summary, anti-inflammatory regulation, angiogenesis, oxidative stress protection, and ECM reconstruction are core biological targets for wound repair, providing a theoretical basis for functional material design and molecular interventions.

### 2.4. Theoretical Basis for Wound Repair by Natural Polysaccharide Hydrogels

Natural polysaccharide hydrogels, constructed from chitosan, hyaluronic acid, sodium alginate, and various functional plant-derived polysaccharides, provide a systemic intervention strategy covering all stages of wound healing through the synergistic integration of material structure design and biological functions. Their theoretical basis is not derived from a single mechanism but is built on a comprehensive framework of multi-bioactivity synergy, precise regulation of key signaling pathways, and structural design that amplifies biological effects, enabling them to exert therapeutic potential against multiple pathological obstacles in chronic and complex wound environments [76].

#### 2.4.1. Synergistic Multi-Bioactivity to Improve the Wound Microenvironment

The essence of impaired wound healing lies in the imbalance of the local microenvironment, including persistent inflammation, accumulation of oxidative stress, insufficient angiogenesis, and restricted cellular behaviors. A prominent advantage of natural polysaccharide hydrogels is their ability to integrate the biological properties of different polysaccharides and functional components, generating synergistic rather than merely additive therapeutic effects, thereby systematically improving the wound microenvironment [36].

At the level of immune regulation, polysaccharide hydrogels can alleviate prolonged inflammation by suppressing pro-inflammatory signaling and guiding macrophage phenotype conversion. Numerous studies have shown that chitosan and plant-derived polysaccharides can intervene in inflammation-related pathways such as NF-κB, reducing the sustained release of pro-inflammatory factors like TNF-α and IL-1β, while promoting the proportion of reparative M2 macrophages, creating favorable conditions for the initiation of the proliferative phase [77].

In oxidative stress regulation, the intrinsic free radical scavenging ability of polysaccharide molecules, combined with nanozymes or antioxidant-active molecules, can effectively reduce ROS-induced damage to keratinocytes, fibroblasts, and endothelial cells, and maintain redox homeostasis by activating endogenous defense pathways such as Nrf2 [78,79,80].

Moreover, natural polysaccharide hydrogels also possess inherent advantages in antibacterial activity and hemostasis. The cationic nature of chitosan, combined with metal ions or photothermal components, can inhibit both free bacteria and biofilm formation, while interactions between the polysaccharide network and blood components facilitate rapid hemostasis and the formation of an initial protective barrier [81].

Through the synergistic effects of these multiple bioactivities, polysaccharide hydrogels can transform the vicious cycle of “inflammation–oxidation–infection–impaired repair” in chronic wounds into a microenvironment conducive to tissue regeneration [37,82,83,84,85,86,87].

#### 2.4.2. Regulation of Cellular Signaling Pathways to Support Repair Mechanisms

The biological effects of natural polysaccharide hydrogels ultimately manifest as modulation of cellular behaviors and tissue regeneration, and their underlying mechanisms are closely associated with multiple key signaling pathways, exhibiting “pathway-specific regulation” characteristics [88]. In inflammation regulation, the NF-κB pathway is a central node persistently activated in chronic wounds. Polysaccharide hydrogels can inhibit NF-κB nuclear translocation by reducing oxidative stress stimuli, interfering with IκBα phosphorylation, or modulating immune cell metabolic status, thereby attenuating pro-inflammatory signaling cascades and facilitating the transition from inflammation to the repair phase [89,90].

During angiogenesis, the HIF-1α/VEGF axis is a key regulatory pathway responding to hypoxia and driving neovascular formation. Polysaccharide hydrogels can stabilize HIF-1α or enhance VEGF signaling, directly promoting endothelial cell migration and lumen formation, while indirectly improving the angiogenic microenvironment by modulating macrophage and fibroblast functions [91]. In oxidative stress protection and cell preservation, the Nrf2 signaling pathway forms the core defense against oxidative damage in wound cells. Polysaccharides and their composites can promote Nrf2 activation and upregulate the expression of antioxidant enzymes such as HO-1 and NQO1, thereby enhancing cellular tolerance to high ROS environments [79,81,92].

Additionally, pathways such as PI3K/Akt play important roles in cell survival, migration, and proliferation. Polysaccharide hydrogels can indirectly activate these pathways by improving ECM structure, providing bioactive cues, and optimizing local nutrient and oxygen supply, thereby restoring fibroblast and keratinocyte function [15]. These signaling pathways do not operate in isolation but form a synergistic regulatory network within the favorable microenvironment created by polysaccharide hydrogels, jointly driving wound repair [78,87].

#### 2.4.3. Structural Design to Enhance Biological Effects and Clinical Adaptability

Beyond the intrinsic bioactivity of the material, the structural design and delivery functionality of natural polysaccharide hydrogels are critical for translating their theoretical advantages into actual therapeutic outcomes. Constructing double or multiple network structures can significantly enhance mechanical stability and adaptive capability, making the hydrogels suitable for irregular, dynamic, or high-stress wound environments. Simultaneously, the introduction of dynamic covalent bonds or physically reversible interactions can impart self-healing and injectability, improving clinical handling flexibility [15,93,94].

On this basis, polysaccharide hydrogels serve as delivery carriers capable of local, controlled release of antibacterial drugs, growth factors, antioxidant molecules, exosomes, nucleic acids, or other bioactive factors. By designing responsiveness to pH, glucose concentration, or redox status, the hydrogels can adjust release behavior according to changes in the wound microenvironment, providing stage-appropriate therapeutic signals at different healing phases. This “structure–function–delivery” integrated design transforms polysaccharide hydrogels from passive dressings into actively regulatory therapeutic platforms [95].

In summary, natural polysaccharide hydrogels, through synergistic multi-bioactivity, regulation of key signaling pathways, and reinforcement via structural and delivery strategies, establish a theoretical framework encompassing all stages of skin wound healing. This framework not only explains their comprehensive advantages in anti-inflammation, antioxidation, angiogenesis, and tissue reconstruction but also provides a clear biological rationale for the discussion of material design strategies and specific application scenarios in subsequent sections.

## 3. Natural Polysaccharide-Based Hydrogels: Material and Engineering Toolbox

In Section 2, the pathological mechanisms underlying impaired skin wound healing and their key biological repair targets were systematically described, and the unique advantages of natural polysaccharide hydrogels in modulating inflammation, oxidative stress, angiogenesis, and tissue reconstruction were theoretically demonstrated. However, the realization of these biological effects does not rely solely on the intrinsic activity of polysaccharides but is highly dependent on the synergistic action of material selection, structural design, and engineering strategies.

Therefore, to transition from “mechanistic understanding” to “practical therapeutic systems”, it is necessary to view natural polysaccharide hydrogels as an engineering toolbox composed of material units, crosslinking methods, and functionalization techniques. Within this toolbox, polysaccharides of different origins and structures provide fundamental biocompatibility and bioactivity, crosslinking and structural design impart mechanical and dynamic properties suitable for the wound environment, and functionalization and composite strategies further amplify their therapeutic effects at specific repair stages.

This chapter focuses on the material basis and engineering realization of natural polysaccharide hydrogels. It first introduces representative natural polysaccharides commonly used for skin repair and their structural features, and then systematically summarizes crosslinking strategies and functional enhancement approaches, providing material and engineering foundations for the design logic in specific application scenarios discussed in subsequent chapters.

### 3.1. Natural Polysaccharide Materials

In summary, natural polysaccharides from different sources possess distinct characteristics: animal-derived polysaccharides emphasize bioactivity and regulation of cellular signaling, plant- and algae-derived polysaccharides focus on hydrogel mechanics and moldability, while microbial and functional polysaccharides provide stability and additional biological functions. Through rational combination, chemical modification, or incorporation of nanocomponents, the mechanical properties, degradability, and biological functionality of hydrogels can be significantly enhanced, laying the foundation for subsequent crosslinking strategies and structural design. Common types of natural polysaccharides and their key features are summarized in Table 1.

Due to their abundant availability, excellent biocompatibility, biodegradability, and low toxicity, natural polysaccharides have been widely applied in wound healing and skin tissue regeneration [96]. They not only provide a basic hydrogel scaffold but can also carry multiple bioactivities (e.g., anti-inflammatory, antioxidant, antibacterial, and pro-angiogenic effects) through structural and functional design, thereby enabling regulation across all stages of wound healing. Natural polysaccharides can be classified into four categories based on their sources: animal, plant, algae, and microbial, each with unique structural features and application advantages.

Polysaccharides of animal origin mainly include chitosan (CS) and hyaluronic acid (HA). Chitosan is the only naturally occurring cationic polysaccharide, exhibiting broad-spectrum antibacterial activity, hemostatic properties, and good film-forming ability; however, its water solubility is limited and can be improved via modifications such as carboxyethylation or glycosylation [97,98,99,100]. Hyaluronic acid is widely present in the dermis, synovial fluid, and vitreous body, exhibiting high hydrophilicity and the ability to modulate cell behavior, promoting angiogenesis and ECM remodeling; however, its monomeric mechanical strength is weak and it is easily degraded, often requiring chemical modification or combination with nanocomponents to enhance performance.

Plant-derived polysaccharides are abundant, including cellulose, starch and their derivatives, as well as arabic gum, guar gum, konjac glucomannan, etc. These polysaccharides generally possess good swelling and rheological properties, which can improve hydrogel formability and mechanical performance [83,101]. Algal polysaccharides such as sodium alginate, carrageenan, and agar exhibit pronounced ionic crosslinking characteristics; for example, sodium alginate can form a stable “egg-box” structure with Ca^2+^, making it suitable for exudate management and drug loading [102]. Microbial polysaccharides, including xanthan gum, gellan gum, dextran, and pullulan, as well as fungal polysaccharides from species such as *Lentinus edodes* (shiitake) and *Ganoderma lucidum* (reishi), generally have controllable molecular weight, good batch-to-batch consistency, and potential bioactivity, and can be produced at scale via fermentation processes [103]. Emerging functional polysaccharides, such as snail mucin glycosaminoglycans (AFG) and konjac glucomannan (KGM), combine biocompatibility with targeted anti-inflammatory activity and pro-angiogenic capacity, and can be incorporated with GelMA or nanoparticles to form multifunctional hydrogels [38].

#### 3.1.1. Chitosan

Chitosan is a natural polysaccharide second only to cellulose in abundance in nature, and it is currently the only known naturally occurring cationic polysaccharide. Its molecular structure consists of N-acetylglucosamine and D-glucosamine units linked via β-(1→4) glycosidic bonds to form a linear chain, widely sourced from crustacean shells, insect exoskeletons, and fungal cell walls [97]. The abundant free amino groups on the chitosan chain can be easily protonated to –NH_3_^+^ under acidic conditions, endowing it with excellent electrostatic interaction capabilities, which form the structural basis for its antibacterial, hemostatic, and cell-adhesive properties.

In wound healing, the advantages of chitosan mainly manifest in three aspects: first, its cationic nature can disrupt bacterial cell membranes to achieve broad-spectrum antibacterial effects and help inhibit biofilm formation; second, chitosan can activate platelets and promote fibrin deposition, accelerating hemostasis and early coagulation responses; third, its good film-forming ability and biocompatibility facilitate the construction of a protective moist microenvironment, promoting fibroblast and keratinocyte adhesion and migration [98]. However, natural chitosan has poor water solubility under neutral conditions and a narrow processing window, limiting its direct application in hydrogel systems. Therefore, chitosan is often functionalized through chemical modification or copolymerization strategies. For example, carboxyethyl chitosan (CEC) can be prepared via Michael addition reaction; the introduction of carboxyl groups significantly improves water solubility and pH stability while retaining partial cationic antibacterial properties [99]. In addition, the Muhammad group constructed targeted nanoparticles via reductive amination of chitosan with mannose, which can specifically recognize and inhibit *Helicobacter pylori*, overcoming its drug resistance [100]. In summary, chitosan and its derivatives, with their combined advantages of “natural antibacterial activity + high modifiability + abundant crosslinking sites”, have become one of the core foundational materials for multifunctional wound hydrogel systems, playing a key role in subsequent crosslinking design and stimuli-responsive construction.

#### 3.1.2. Hyaluronic Acid

Hyaluronic acid (HA) is a linear acidic glycosaminoglycan composed of alternating D-glucuronic acid and N-acetyl-D-glucosamine units linked by β-1,3 and β-1,4 glycosidic bonds, widely present in the dermis, synovial fluid, and vitreous body [104]. By binding to cell surface CD44 receptors, HA regulates fibroblast and endothelial cell proliferation and migration, promotes collagen deposition and angiogenesis, and modulates inflammatory responses to reduce wound swelling [83]. As an essential component of the extracellular matrix (ECM), HA plays a key role in maintaining tissue hydration, buffering mechanical stress, and regulating cell behavior [105]. During wound repair, HA mainly acts by interacting with cell surface receptors such as CD44 to promote the migration and proliferation of keratinocytes, fibroblasts, and endothelial cells, while modulating inflammation and promoting angiogenesis. Its high hydrophilicity allows it to retain large amounts of water, providing a stable moist environment that facilitates granulation tissue formation and re-epithelialization [106]. However, natural HA has low mechanical strength and is easily degraded by hyaluronidase in vivo, making it insufficient for complex or chronic wound applications when used alone. Therefore, HA is often chemically modified (e.g., oxidation, hydrazide modification, methacrylation) or combined with nanofibers, proteins, or other polysaccharides to construct hydrogels with self-healing, injectable, or controllable degradation properties. Overall, HA in natural polysaccharide systems tends to function as a “bio-signal regulating and cell-guiding material,” usually combined with other polysaccharides to endow hydrogels with migration-promoting, angiogenic, and anti-inflammatory properties [107].

#### 3.1.3. Sodium Alginate

Sodium alginate (SA) is a natural anionic polysaccharide derived from brown algae, composed of β-D-mannuronic acid (M) and α-L-guluronic acid (G) residues linked by 1→4 glycosidic bonds. The abundant carboxyl groups on its chain provide excellent hydrophilicity and ionic crosslinking capability, making it one of the earliest natural polysaccharides systematically used for hydrogel construction.

The most representative feature of SA is its ability to form stable ionic crosslinked networks with multivalent metal ions such as Ca^2+^ and Fe^2+^ via the “egg-box model”. This process occurs under mild conditions without the need for organic crosslinkers, suitable for loading active proteins, cells, or drugs [105]. In wound repair, SA hydrogels possess good absorptive capacity and wound adhesion, making them suitable for highly exuding or infected wound management. However, single-component SA hydrogels have limited mechanical strength, relatively simple functionality, and lack intrinsic antibacterial activity. Recently, the introduction of metal ions, antibacterial agents, or chemical grafting modifications has significantly improved their antibacterial, adhesive, and mechanical properties [86,108]. Therefore, SA in polysaccharide hydrogel systems often serves as a “structural backbone and ion-responsive module,” synergistically constructing multi-layered networks with other polysaccharides or functional components.

#### 3.1.4. Starch

Starch is a natural high-molecular-weight polysaccharide composed of α-D-glucose units linked via α-(1→4) and α-(1→6) glycosidic bonds, mainly including amylose and amylopectin, and widely present in corn, potato, cassava, and other plants [109]. Its broad availability, low cost, and safe processing make it promising for biomedical applications.

In wound repair, starch advantages include its good hydrophilicity and biodegradability, enabling the formation of a moist environment and participation in hydrogel network construction [110]. However, natural starch has poor mechanical properties, is brittle, and degrades relatively quickly, making it difficult to meet the requirements of tissue engineering and complex wound healing alone [111]. Therefore, starch is often modified through oxidation, esterification, or blending with other polymers to form double-network or composite hydrogels, improving mechanical performance and introducing functional sites [112,113,114]. Overall, starch functions primarily as an “economical structural supplement material” in composite systems, regulating gel mechanics and swelling behavior.

#### 3.1.5. Cellulose

Cellulose is the most abundant natural polysaccharide in nature, composed of D-glucose units linked by β-(1→4) glycosidic bonds to form a highly crystalline linear structure [115]. Its excellent mechanical properties, biocompatibility, and stability have attracted attention in tissue engineering and biomedical materials [116]. However, strong intermolecular hydrogen bonding makes cellulose poorly soluble and difficult to process. To expand its applications, researchers often prepare nanocellulose or perform carboxymethylation, hydroxypropylation, or other modifications to improve solubility and reactivity [117]. Modified cellulose can serve as a high-strength network support material, significantly enhancing hydrogel mechanical performance and structural stability [87,118,119,120]. Thus, cellulose and its derivatives primarily act as a “mechanical reinforcement and structural support module” in polysaccharide hydrogel systems, particularly suitable for load-bearing or long-term application materials.

#### 3.1.6. Dextran

Dextran is a microbial polysaccharide connected by α-1,6 and α-1,3 glycosidic bonds, exhibiting good biocompatibility, biosafety, and reactivity [121,122]. Its molecular chains can be easily functionalized with aldehyde or hydroxyl groups, making it suitable for constructing dynamic covalent crosslinked networks. In wound healing, dextran is commonly used to construct injectable, self-healing, and stimuli-responsive hydrogels that promote tissue regeneration by regulating drug release, anti-inflammatory responses, and angiogenesis [123,124,125,126]. Overall, due to its high modifiability, dextran has become an important “dynamic crosslinking and drug delivery carrier” in smart hydrogel design.

#### 3.1.7. Other Natural Polysaccharide Materials

In recent years, emerging polysaccharides from special sources or extensively functionalized, such as snail mucin glycosaminoglycans and konjac glucomannan, have gradually entered the field of wound repair research [38,127]. These polysaccharides generally possess good biocompatibility and unique bioactivities, significantly improving complex wound microenvironments through immune modulation, angiogenesis promotion, or microbiome regulation. The introduction of emerging polysaccharides not only enriches the natural polysaccharide hydrogel material library but also provides new design strategies for multifunctional integrated hydrogels, particularly suitable for precise intervention in infected or chronic non-healing wounds.

In summary, natural polysaccharides from different sources exhibit significant differences in molecular composition, functional group distribution, and physicochemical behavior, which determine their functional emphasis in antibacterial protection, angiogenesis modulation, immune microenvironment remodeling, and mechanical support. Rational selection and combination of polysaccharide types can partially achieve targeted intervention in key biological processes of wound healing. However, the intrinsic properties of a single material are often insufficient to simultaneously meet the comprehensive requirements of mechanical stability, dynamic adaptability, and bioactivity for complex wounds. The full potential of these materials heavily depends on fine control of crosslinking methods and network structures. Therefore, crosslinking strategies and structural construction are not only technical means for forming natural polysaccharide hydrogels but also the core engineering tools linking material properties to biological functions.

The common types of natural polysaccharides are detailed in Table 1.

**Table 1:** Properties and Applications of Representative Natural Polysaccharide Hydrogels.

Natural Polysaccharide	Source	Key Functions	Mechanism of Action	Typical Applications	Representative References
Chitosan (CS)	Crustacean exoskeletons, fungal cell walls	Rapid hemostasis, antibacterial, immunomodulation	Positively charged amino groups bind to red blood cells/platelets to promote coagulation; disrupt bacterial membranes; regulate macrophage M1→M2 polarization	Acute wounds, burns, surgical incisions	[128,129]
Sodium Alginate (SA)	Marine algae	Hemostasis, exudate absorption, moist wound healing environment	Forms an “egg-box” network with Ca^2+^; high water absorption and swelling; maintains a moist microenvironment	Acute wounds, moist wound dressings	[130]
Hyaluronic Acid (HA)	Human tissues (e.g., skin, synovial fluid), microbial fermentation	Promotes re-epithelialization, angiogenesis, anti-inflammatory	Regulates keratinocyte migration; promotes M2 macrophage polarization; can deliver drugs/VEGF/growth factors	Full-thickness skin defects, chronic ulcers	[4,131,132]
Gelatin (GEL)	Hydrolysis of animal collagen	Angiogenesis, cell adhesion, scaffold formation	Mimics the natural extracellular matrix (ECM), supports fibroblast and endothelial cell adhesion and proliferation	Skin tissue engineering scaffolds, chronic wounds	[133]
Chitosan-Gelatin Composite (CS/GEL)	Composite of chitosan and gelatin	Integrated mechanical reinforcement, angiogenesis, immunomodulation	Multi-network/gradient structure provides mechanical support and energy dissipation; composite network promotes vascularization	Skin tissue engineering, wound repair	[134]
*Bletilla striata* Polysaccharide (BSP)	Tubers of *Bletilla striata*	Anti-biofilm, anti-inflammatory, promotes healing	Inhibits bacterial biofilm formation; modulates inflammatory cytokines; promotes fibroblast proliferation	Diabetic foot ulcers, chronic wounds	[135]
Berberine-BSP Composite Hydrogel	Composite of BSP and berberine	Antibacterial, anti-inflammatory, promotes angiogenesis	Berberine provides antibacterial/anti-inflammatory effects; BSP provides a 3D scaffold structure to promote vascularization	Chronic ulcers, pressure sores	[135]
HPCS-C (Hydroxypropyl Chitosan-Catechol Conjugate)	Chemically modified chitosan	Injectable, self-healing hydrogel, rapid hemostasis	Dynamic Schiff base cross-linking imparts self-healing properties; rapidly forms a hemostatic barrier	Acute wounds, non-compressible wounds	[128]
Polysaccharide-Nanoparticle Composite Hydrogel	Composite of polysaccharides and nanoparticles (e.g., silver nanoparticles)	Antibacterial, antioxidant, intelligent drug delivery	Nanoparticles enable controlled release of silver ions or drugs; polysaccharide network provides a moist environment	Chronic wounds, infected wounds	[20,130,136]
Polysaccharide-Protein Composite Hydrogel	Composite of polysaccharides and proteins (e.g., collagen)	Mechanical reinforcement, angiogenesis, re-epithelialization	Multi-network structure enhances toughness and self-healing; proteins provide cell adhesion sites	Skin tissue engineering, full-thickness skin defects	[133,137]

### 3.2. Crosslinking Strategies and Key Characterization Techniques

In the construction of natural polysaccharide-based hydrogels, the crosslinking method is a key factor that determines the network structure and macroscopic properties. Different crosslinking strategies can significantly influence the hydrogel’s pore structure, mechanical performance, swelling and degradation behavior, as well as biological activity by regulating the types of intermolecular interactions and crosslinking density [138]. Overall, physical crosslinking typically offers mild reaction conditions and avoids residual chemical reagents, making it suitable for applications that demand high biocompatibility [139]. Chemical crosslinking, on the other hand, allows precise control over the type of crosslinker and reaction conditions, enabling targeted modulation of hydrogel mechanical strength and structural stability to meet specific application requirements [140]. With advances in materials science, novel crosslinking technologies such as click chemistry and enzyme-catalyzed crosslinking have gradually been introduced into natural polysaccharide hydrogel systems, further expanding their functional design space and application scope [141].

#### 3.2.1. Physical Crosslinking

Physical crosslinking primarily relies on non-covalent interactions between molecules, including hydrogen bonding, hydrophobic interactions, and ionic interactions. This type of crosslinking usually occurs under mild conditions, avoiding the introduction of organic crosslinkers and ensuring good biocompatibility. By adjusting polysaccharide concentration, ionic strength, or external environmental conditions, the gel strength and swelling behavior of the hydrogel can be modulated within a certain range; however, the network structure is relatively loose, and mechanical stability and long-term durability are limited. Therefore, physically crosslinked hydrogels are often used in wound applications that require injectability, sprayability, and rapid gelation.

##### Hydrogen Bond Interactions

Hydrogen bonds are among the most common forms of physical interactions, usually formed through electrostatic interactions between hydrogen donors and highly electronegative atoms (such as N, O, or F) [142]. Due to their relatively low bond energy, hydrogen bonds can break and reform rapidly, endowing materials with dynamic adaptability [143].

Zhao, Jing et al. [144] constructed a continuous hydrogen-bond network to prepare ultra-stretchable starch-based hydrogels. FTIR spectra showed a broadened –OH stretching peak around 3200 cm^−1^, confirming the formation of dense hydrogen bonds; SEM images further revealed a porous structure (pore size ~50–200 μm), which is conducive to cell migration and nutrient diffusion. Feng Jiang’s group [145] proposed a multiscale hydrogen-bond reinforcement strategy by introducing monosaccharides at the molecular level and nano/microscale polysaccharides to build a cross-scale hydrogen-bond network, significantly enhancing hydrogel toughness and mechanical stability, providing new ideas for the design of high-performance natural polysaccharide hydrogels [145].

##### Hydrophobic Interactions

Hydrophobic interactions refer to the aggregation of hydrophobic groups in aqueous environments to form three-dimensional network structures. When external forces disrupt the network, hydrophobic aggregates can rapidly reorganize through molecular rearrangement [146]. This type of crosslinking generally imparts hydrogels with self-healing behavior, shear-thinning properties, and enhanced mechanical stability [147]. Tao Wang [148] constructed a thermosensitive hydrogel system using chitosan, hydroxypropyl methylcellulose, and glycerol under physiological conditions. Hydroxypropyl methylcellulose promoted thermal gelation through hydrophobic interactions, while high glycerol concentrations disrupted the polymer hydration layer to enhance hydrophobic domain formation, lowering the phase transition temperature. The hydrogel gelled at approximately 37 °C within 15 min, exhibiting good fluidity, thermosensitivity, biodegradability, low cytotoxicity, and certain controlled-release capabilities, showing potential for biomedical applications [149].

##### Ionic Interactions

Ionic crosslinking is formed through reversible electrostatic interactions between oppositely charged ions or polymer chains [150]. This type of crosslinking occurs under mild and controllable conditions and exhibits good biocompatibility, widely applied in biomedical fields [151]. Typical systems include sodium alginate (SA) crosslinked with divalent metal ions such as Ca^2+^ via the “egg-box” model, as well as ionic interactions between cationic amino groups on chitosan chains and polyanionic crosslinkers [152,153]. Lili Wang [154] activated latent interaction sites among SA chains through a “free water evaporation” strategy and introduced Ca^2+^ to construct a fully physically crosslinked network with “hydrogen bond–ionic bond synergistic reinforcement,” where ionic bonds provided the main mechanical support. Yanjun Yang et al. [155] used a neutral polysaccharide GP1-2 isolated from licorice and, under alkaline conditions, coordinated Ca^2+^ with hydroxyl and ether oxygen groups to construct a porous physically crosslinked hydrogel, providing spatial capacity for drug loading. Due to their responsiveness to ionic strength and environmental pH, ionically crosslinked hydrogels show great potential in drug-controlled release, cell encapsulation, and wound dressing, and the mild formation conditions help preserve bioactive molecules and cell viability [133].

#### 3.2.2. Chemical Crosslinking

Chemical crosslinking involves the reaction between active groups on polysaccharide chains and crosslinkers, forming stable covalent bonds and constructing three-dimensional networks [156]. Compared with physical crosslinking, chemical crosslinking can significantly enhance the hydrogel’s mechanical strength, thermal stability, and chemical stability. By controlling the type and amount of crosslinker and reaction conditions, the crosslinking density, pore structure, and degradation behavior can be precisely modulated [141]. Therefore, chemical crosslinking is widely applied in tissue engineering scaffolds and long-term drug delivery systems, although the biocompatibility and potential residual toxicity of crosslinkers must be considered [157].

##### Schiff Base (Imine) Crosslinking

Imine bonds are one of the most common dynamic reversible covalent bonds, usually formed by nucleophilic addition between aldehyde or ketone groups and primary amines. The reaction conditions are mild and proceed rapidly in aqueous solutions, allowing hydrogels to maintain covalent network stability while exhibiting self-healing and injectability [158]. For example, Shangzhi Li et al. [159] introduced aldehyde groups via oxidized hyaluronic acid and reacted them with cystamine dihydrochloride to construct a dynamic crosslinked network. Changyuan He et al. [160] used oxidized hyaluronic acid and adipic dihydrazide-modified HA to form hydrazone networks, loading polydopamine nanoparticles to achieve photothermal antibacterial effects and NO release under near-infrared light. Based on its dynamic and reversible properties, Schiff base crosslinking provides an important platform for designing smart hydrogels with mechanical adaptability and stimuli-responsiveness.

##### Boronate Ester Crosslinking

Boronate ester crosslinking is based on the reversible reaction between boronic acid groups and cis-diols to form dynamic covalent networks, offering good environmental responsiveness and biocompatibility, commonly used in self-healing hydrogel construction [161]. Yonghang Liu et al. [162] designed the QL@MAB hydrogel, where hydrophobic MA segments formed a dense surface layer, and AAPBA-boronate ester bonds with polyphenolic drugs triggered structural reconstruction and drug release under hyperglycemic conditions, accelerating diabetic wound healing. Yang Yu et al. [163] grafted 3-carboxyphenylboronic acid onto chitosan and PVA to construct a pH-responsive self-healing hydrogel with anti-tumor recurrence and antibacterial capabilities. In summary, boronate ester crosslinking strategies provide strong support for chronic wound management and smart medical material design [162].

##### Epoxy Crosslinking

Epoxy crosslinking involves the ring-opening reaction of epoxy compounds with hydroxyl or amino groups on polysaccharide chains, forming stable covalent ether or secondary amine bonds with high structural stability and mechanical strength [103]. Pingdong Wei et al. [164] used epichlorohydrin as a crosslinker to prepare highly elastic chemically crosslinked hydrogels and further induced physical crystallization via ethanol vapor to construct a dual-network system (“epoxy covalent crosslinking–physical crystallization crosslinking”). This design enhances mechanical performance and structural remodeling capacity, providing new ideas for high-performance cellulose-based hydrogels. Epoxy-crosslinked hydrogels are particularly valuable in tissue engineering scaffolds requiring long-term service due to their excellent structural stability.

##### Free Radical Polymerization Crosslinking

Free radical polymerization crosslinking uses light- or heat-initiated chain polymerization of polysaccharide derivatives containing unsaturated double bonds to form covalent networks. This method offers rapid reaction rates and high crosslinking efficiency, allowing precise control over gelation kinetics and mechanical properties by adjusting light exposure or initiator concentration [165]. Yaqin Li et al. [166] polymerized acrylamide monomers in the presence of carboxymethyl chitosan nanoparticles and copper ions to construct hydrogels with antibacterial, hemostatic, and pro-healing functions. Although free radical polymerization offers advantages in mechanical property tuning, clinical translation requires attention to unreacted monomers and residual initiators affecting biocompatibility.

In summary, after systematically reviewing the intrinsic properties of natural polysaccharides and their hydrogel crosslinking mechanisms, it can be concluded that natural polysaccharides, due to their abundant sources, tunable physicochemical properties, and excellent biological performance, are ideal materials for constructing functionalized hydrogel systems [167]. Natural polysaccharides include animal-derived chitosan and hyaluronic acid, plant-derived cellulose and starch, as well as alginate and microbial fermentation polysaccharides. Each polysaccharide has unique properties, which can be effectively enhanced and expanded through molecular modification and functionalization [168].

Simultaneously, diverse crosslinking strategies provide rich options for tuning the properties of natural polysaccharide hydrogels. These strategies include physical crosslinking (such as hydrogen bonding, hydrophobic interactions, and ionic interactions) and chemical crosslinking (such as Schiff base, boronate ester, epoxy, and free radical polymerization) [141]. Physical crosslinking preserves bioactivity due to mild conditions, chemical crosslinking significantly improves mechanical strength and stability, while dynamic covalent crosslinking combines stability and reversibility, imparting self-healing and stimuli-responsive features [169].

**Figure 3:**
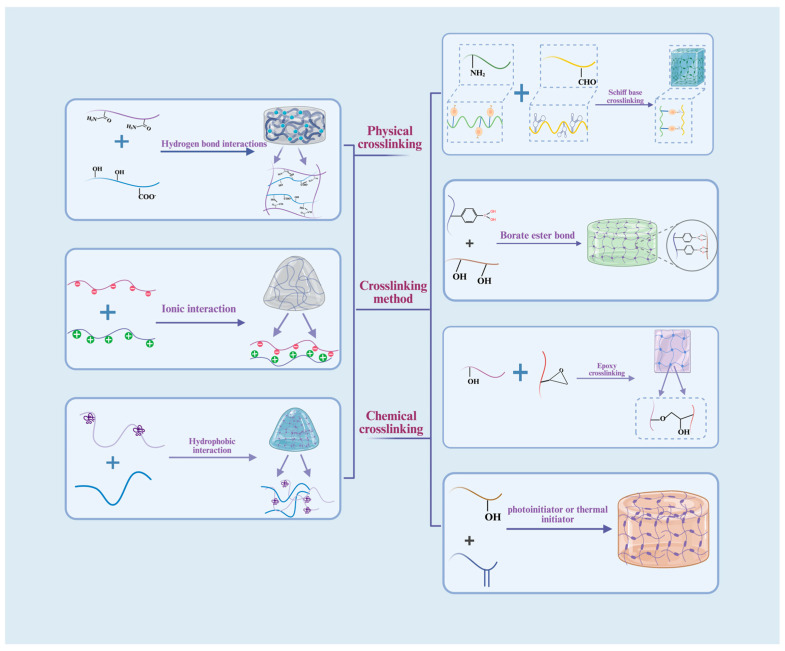
Schematic Illustration of the Structure and Crosslinking Mechanisms of Natural Polysaccharide Hydrogels.

The research on these crosslinking mechanisms provides a solid theoretical foundation for the application of natural polysaccharide hydrogels in skin tissue engineering and wound repair. By rationally selecting polysaccharide types and crosslinking strategies, high-performance hydrogel dressings and tissue engineering scaffolds tailored to specific clinical needs can be designed, opening broad prospects for translational applications. Figure 3 schematically illustrates the representative molecular structures of natural polysaccharides and the corresponding physical and chemical crosslinking mechanisms that govern hydrogel network formation, providing a structural foundation for subsequent network topology engineering and multifunctional design.

### 3.3. Structural Design and Functional Enhancement Strategies

After clarifying the materials and crosslinking foundations of natural polysaccharide hydrogels, performance enhancement has shifted toward a new stage of multiscale structural engineering and functional synergy. By systematically designing network topology, microstructure, and functional units, the mechanical properties, bioactivity, and interactions with the wound microenvironment can be co-regulated across multiple dimensions, thereby promoting a shift from “passive adaptation” to “active modulation.”

The main enhancement strategies include:

(1) Network topology engineering, such as constructing double networks, interpenetrating networks, or gradient networks, to synergistically enhance strength, toughness, and dynamic adaptability;
(2) Spatial biomimetic configurations, precisely controlling pore size, pore connectivity, and anisotropy to guide cell migration, angiogenesis, and tissue reconstruction;
(3) Functional module integration, introducing antibacterial, antioxidant, and immunomodulatory components to establish multi-mechanism therapeutic systems;
(4) Coupled smart responsiveness and delivery, combining stimulus-responsive behaviors to achieve spatiotemporally precise release of drugs or bioactive factors.

These strategies are closely linked to crosslinking mechanisms: dynamic crosslinking provides a basis for self-healing and responsiveness, multi-network structures enhance mechanical stability, and porous and gradient architectures optimize mass transport and cell interactions. This “crosslinking–structure–function” integrated design promotes natural polysaccharide hydrogels toward programmable, highly adaptive next-generation wound healing platforms.

#### 3.3.1. Multi-Network Topology: Synergy of Mechanical Enhancement and Dynamic Adaptability

Single-network natural polysaccharide hydrogels often struggle to simultaneously meet the requirements of high mechanical strength, flexibility, and dynamic adaptability, especially under wound stretching, fluid flow, or tissue motion, which can lead to structural failure. To address this issue, multi-network structural design strategies have been widely adopted. By constructing synergistic structures composed of networks with different crosslinking types or scales within the same system, a balance between mechanical enhancement and energy dissipation can be achieved [170].

Typical double-network (DN) hydrogels generally consist of a highly crosslinked rigid network and a low-crosslinked flexible network: the former provides fundamental mechanical support, while the latter undergoes reversible breakage under external forces to dissipate energy, significantly improving toughness and fatigue resistance [171]. Furthermore, by introducing dynamic covalent bonds (such as Schiff base or boronate ester bonds) into multi-network systems, hydrogels gain self-healing and reconfigurable abilities, maintaining overall structural integrity after repeated deformation or local damage. Such multi-network structures demonstrate distinct advantages in applications requiring high mechanical stability, such as burn wounds or chronic ulcers.

#### 3.3.2. Spatial and Pore Structure Control: Constructing Biomimetic Microenvironments

Beyond network connectivity, the spatial structural features of hydrogels are also critical determinants of biological performance. Pore size, porosity, and pore distribution directly affect oxygen and nutrient diffusion, metabolite removal, and cell adhesion and migration, thereby regulating the efficiency and quality of tissue regeneration.

Porous hydrogels fabricated via freeze-drying, phase separation, or ion-induced crosslinking can provide three-dimensional growth spaces for fibroblasts, endothelial cells, and immune cells, facilitating granulation tissue formation and angiogenesis [172]. Moreover, considering the layered structure of skin tissue, researchers have further designed gradient pore structures or anisotropic hydrogels, enabling different depth regions to meet both barrier protection and tissue regeneration needs. Such precise control of spatial structures brings natural polysaccharide hydrogels closer to the native extracellular matrix at the structural level, providing a physical basis for functional repair of complex wounds [173].

#### 3.3.3. Functional Module Integration: Multi-Target Synergistic Therapy

The pathological process of complex wounds often involves multiple factors such as bacterial infection, inflammatory imbalance, enhanced oxidative stress, and insufficient angiogenesis, making single-function hydrogels insufficient for effective intervention. Based on a modular design concept, researchers have orderly integrated different biofunctional units into natural polysaccharide hydrogel networks to construct composite systems capable of multi-target synergistic regulation [174].

Common functional modules include antibacterial modules (e.g., cationic polysaccharides, metal ions, or photothermal components), antioxidant and ROS-scavenging modules, immunomodulatory modules, and pro-angiogenic modules [172]. These functional units can be incorporated into hydrogel networks through physical loading, chemical grafting, or dynamic bonding to achieve synergistic effects in the local microenvironment. For instance, coupling antibacterial and immunomodulatory functions helps rapidly eliminate pathogens and shorten inflammation duration, while pro-angiogenic modules further promote nutrient supply and tissue reconstruction. Through systematic functional module integration, natural polysaccharide hydrogels gradually transition from “passive dressings” to “actively regulated therapeutic platforms.”

#### 3.3.4. Coupled Delivery and Stimulus-Responsive Design: Spatiotemporally Precise Therapeutic Intervention

To meet the demands of precision therapy, natural polysaccharide hydrogels have evolved from static drug-loading systems to intelligent delivery platforms. By combining structural design with stimulus-responsive mechanisms, controlled release of drugs, bioactive molecules, or cells can be achieved within specific time windows and pathological microenvironments [170].

Stimuli such as temperature, pH, ROS, glucose, and light have been widely employed to construct intelligent hydrogel systems. Thermosensitive hydrogels can rapidly gel in situ after spraying or injection, suitable for irregular wounds [175]; ROS- or glucose-responsive hydrogels enable on-demand release in chronic inflammatory or hyperglycemic environments. Dynamic covalent crosslinking and reversible physical interactions provide the structural basis for these responses, allowing hydrogels to maintain overall stability while adapting to the environment, thereby significantly enhancing therapeutic precision and safety [176]. Regarding sprayable delivery, a clinically relevant wound-adaptive form, Tan et al. [41] systematically characterized the structure, chemical composition, mechanical properties, self-healing, and wound adaptability of sprayable polysaccharide-based hydrogels through optical imaging, SEM, FTIR, rheology, and functional assays, providing robust experimental evidence for their application in skin wound repair. The combination of these characterization techniques and functional validation not only confirms the “structure–function” compatibility of polysaccharide hydrogels but also lays the technical foundation for their application in diverse scenarios, such as rapid coverage of acute wounds or precise delivery to chronic wounds.

In summary, the structural design and functional enhancement of natural polysaccharide hydrogels have evolved from single-material modification to multilevel, programmable engineering systems. By rationally coupling multi-network structures, spatial configurations, functional module integration, and stimulus-responsive mechanisms, synergistic optimization of mechanical performance, bioactivity, and therapeutic behavior can be achieved, providing systematic design strategies for complex wound repair and skin tissue regeneration, and laying a solid foundation for clinical translation [174].

Beyond laboratory-scale material design, natural polysaccharide-based hydrogels have rapidly advanced toward translational and clinical applications, as evidenced by a growing number of patent filings worldwide. These patents primarily focus on composite network construction, rapid gelation strategies, bioactive factor delivery, and multifunctional wound dressings targeting chronic and infected wounds. Representative patented technologies in this field are summarized in Table 2, highlighting key material systems, crosslinking concepts, and their intended therapeutic applications.

## 4. Applications of Natural Polysaccharide Hydrogels in Skin Regeneration

After systematically reviewing the physicochemical properties, crosslinking mechanisms, and functionalization strategies of natural polysaccharide hydrogels, collective evidence indicates that these tunable and biocompatible materials exhibit substantial potential in skin regeneration.

Skin regeneration involves diverse clinical scenarios, including acute wound repair, chronic non-healing wounds, tissue engineering–based reconstruction, and scar modulation, each characterized by distinct pathological features and repair requirements. Nevertheless, natural polysaccharide hydrogels can be rationally engineered through material combinations, functional modifications, and structural optimization to address the key biological challenges associated with different wound types.

**Figure 4:**
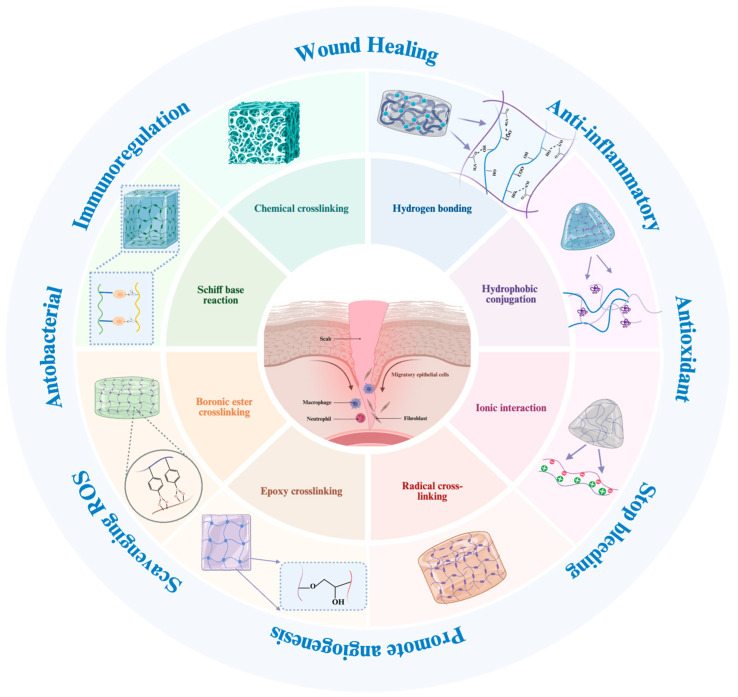
Roles of Various Natural Polysaccharide Hydrogels in Skin Tissue Regeneration.

As illustrated in Figure 4, natural polysaccharide-based hydrogels participate in skin tissue regeneration in a stage-specific and synergistic manner by modulating inflammation, promoting cell migration and proliferation, enhancing angiogenesis, and facilitating extracellular matrix remodeling.

### 4.1. Acute Wound Healing (Burns and Traumatic Injuries)

Acute wounds, such as burns, mechanical injuries, and surgical incisions, are typically characterized by disruption of the tissue barrier, bleeding, high infection risk, and intense inflammatory responses.

An ideal wound dressing should rapidly form a physical barrier, control bleeding, prevent infection, and manage wound exudate, thereby creating a favorable environment for cell migration, proliferation, and tissue remodeling.

Owing to their high water absorption capacity, rapid gelation behavior, and porous network structures, natural polysaccharide hydrogels can form a moist protective barrier on the wound surface, reduce fluid loss, and provide initial hemostatic support.

#### 4.1.1. Rapid Hemostasis and Barrier Formation

Rapid hemostasis is the primary objective in acute wound management. Chitosan-based hydrogels are particularly effective in this regard, as the positively charged amino groups along their polymer chains can interact electrostatically with negatively charged erythrocyte membranes, thereby promoting red blood cell and platelet aggregation while activating the coagulation cascade and accelerating fibrin clot formation.

For example, Rui Tian et al. [128] developed an HPCS-C/ODEX/CC hydrogel composed of catechol-modified hydroxypropyl chitosan (HPCS-C), oxidized dextran (ODEX), and clinoptilolite carbonate (CC) nanoparticles.

This hydrogel exhibited excellent injectability and self-healing properties due to the dynamic Schiff-base bonds between HPCS-C and ODEX, enabling rapid formation of a hemostatic barrier even in complex wound geometries.

Similarly, Shen Guo’s group [129] fabricated an injectable hydrogel based on dynamic ionic interactions and hydrogen bonding between quaternized chitosan (QCS) and tannic acid (TA), which achieved rapid hemostasis in mouse tail amputation, femoral artery hemorrhage, and liver incision models, while significantly accelerating full-thickness skin wound healing [178,181].

In addition, sodium alginate hydrogels, through their ionically crosslinked “egg-box” structures formed between G blocks and Ca^2+^ ions, can rapidly seal wounds, accelerate coagulation, and provide a moist healing environment, thereby markedly promoting the healing process.

#### 4.1.2. Antimicrobial Activity and Inflammation Modulation

Wound infection leads to sustained inflammation and severely impairs the healing process.

Natural polysaccharide hydrogels provide multifunctional solutions through intrinsic antibacterial activity, intelligent drug delivery, and immunomodulation of the wound microenvironment.

Cationic polysaccharides, such as chitosan and its derivatives, can disrupt bacterial membranes via electrostatic interactions, ultimately leading to bacterial cell death [182] Chenrong Wang et al. [183] developed HTCC/PVA composite nanofibrous membranes that exhibited strong contact-killing antibacterial activity against *Staphylococcus aureus* and *Escherichia coli*. Hydrogels loaded with silver nanoparticles (AgNPs) or Zn^2+^ ions enable sustained antimicrobial release and exhibit long-term inhibitory effects against drug-resistant bacteria and *Pseudomonas aeruginosa* [130]. Moreover, hydrogels incorporating photothermal agents such as polydopamine can physically disrupt bacterial biofilms under near-infrared irradiation, achieving effective photothermal sterilization [184].

Meanwhile, hydrogels can regulate immune cell behavior through both chemical composition and physical architecture. Hyaluronic acid (HA) hydrogels have been shown to induce macrophage polarization from the pro-inflammatory M1 phenotype toward the pro-regenerative M2 phenotype [185]. Wanglin Duan et al. [132] developed an HA-DA/PRP-based dressing that exhibited antibacterial activity, promoted granulation tissue regeneration and angiogenesis, and modulated M1/M2 macrophage polarization, thereby accelerating wound healing.

#### 4.1.3. Promotion of Re-Epithelialization and Tissue Reconstruction

During the proliferative phase of wound healing, the formation of granulation tissue and re-epithelialization represent the central reparative events. The three-dimensional porous structure of natural polysaccharide hydrogels provides a favorable scaffold for fibroblast and endothelial cell migration and proliferation [177,179]. Hyaluronic acid (HA), for instance, can directly promote keratinocyte migration and proliferation, significantly accelerating the re-epithelialization process. In addition, smart hydrogels loaded with vascular endothelial growth factor (VEGF) or designed to stabilize hypoxia-inducible factor-1α (HIF-1α) via deferoxamine (DFO) enable sustained, localized angiogenic stimulation, thereby supplying sufficient oxygen and nutrients to regenerating tissues [133,179].

### 4.2. Chronic Wound Healing (Diabetic Foot Ulcers and Pressure Ulcers)

Chronic wounds, such as diabetic foot ulcers and pressure ulcers, involve complex pathological mechanisms, including persistent inflammation, bacterial biofilm formation, impaired angiogenesis, and cellular dysfunction.

Recent studies have demonstrated that natural polysaccharide hydrogels, owing to their unique biological activities and highly tunable physicochemical properties, offer significant advantages in the treatment of chronic wounds.

#### 4.2.1. Anti-Biofilm Activity and Immune Regulation

Biofilm formation and chronic inflammation are key factors contributing to delayed healing in chronic wounds. For example, Xingcan Chen et al. [135] developed a self-healing composite hydrogel based on *Bletilla striata* polysaccharide (BSP), berberine (BER), and borax via borate ester bonds. This hydrogel significantly promoted wound healing in diabetic ulcer (DU) mouse models, achieving a healing rate of up to 94%. In addition to exhibiting antibacterial inhibition rates exceeding 90% against *E. coli* and *S. aureus*, the hydrogel markedly reduced nitric oxide (NO) levels, thereby exerting anti-inflammatory effects. Furthermore, Xueling Liu et al. [180] developed a hybrid hydrogel dressing that functioned as an artificial bio-adhesive barrier at the wound site, maintaining local microbial and immune homeostasis while providing effective anti-inflammatory activity.

#### 4.2.2. Oxidative Stress Regulation and Angiogenesis

Excessive oxidative stress in chronic wounds severely hinders the healing process.

Pressure ulcers are a common clinical problem in elderly individuals and patients with impaired mobility.

Junjie Tang and colleagues [186] developed a thermosensitive injectable hydrogel (adEHG) by combining gallic acid–modified hydroxybutyl chitosan (HBC-GA) with soluble acellular extracellular matrix (adECM).

This hydrogel promoted angiogenesis, cell proliferation, and collagen deposition, while further enhancing inflammation regulation and wound healing through sustained release of therapeutic factors and cells.

### 4.3. Skin Tissue Engineering Scaffolds

Skin tissue engineering aims to construct bioactive skin substitutes for the repair of full-thickness skin defects.

Natural polysaccharide hydrogels have emerged as ideal scaffold materials due to their extracellular matrix–like three-dimensional network structures, excellent biocompatibility, and tunable physicochemical properties.

#### 4.3.1. Biomimetic Structure and Regulation of Cellular Behavior

An ideal skin tissue engineering scaffold should mimic the physicochemical characteristics of the native extracellular matrix to support cell adhesion, proliferation, and differentiation. Kun Li et al. [134] developed a chitosan/gelatin composite hydrogel with anisotropic fibrous structures that effectively recapitulated the topological features of native dermal extracellular matrix.

The composite hydrogel exhibited low immunogenicity, thin fibrous capsule formation, enhanced angiogenesis, and an increased M2/M1 macrophage ratio.

This polysaccharide–protein composite hydrogel provides a novel scaffold material for tissue engineering by promoting vascularization and establishing a favorable immunological microenvironment for tissue repair.

#### 4.3.2. Vascular Network Construction

The establishment of functional vascular networks remains a critical challenge in the regeneration of large full-thickness skin defects.

Yuhan Wang et al. [136] developed a tissue-engineered scaffold composed of gelatin methacrylate (GelMA), oxidized hyaluronic acid (OHA), carboxymethyl chitosan (CMCS), and 2-methacryloyloxyethyl phosphorylcholine (MPC) for skin defect repair.

The incorporation of zwitterionic MPC endowed the bioink with electrical signaling properties similar to those of native skin tissue.

By integrating human foreskin fibroblasts (HFF-1), human umbilical vein endothelial cells (HUVECs), and immortalized human keratinocytes (HaCaTs), a bilayer conductive skin scaffold consisting of an epidermal layer and a vascularized dermal layer was constructed.

In vivo studies demonstrated that the conductive scaffold provided an appropriate electrical microenvironment for cell signaling, growth, migration, and differentiation, ultimately accelerating re-epithelialization, collagen deposition, and vascularization.

#### 4.3.3. Dynamic Mechanical Property Regulation

The mechanical properties of scaffolds play a crucial role in skin development and regeneration.

Qing Liu and colleagues [137] designed a series of self-healing conductive hydrogels based on carboxymethyl chitosan, oxidized sodium alginate, poly(thiosulfate), and iron ions for the treatment of infected wounds.

The dynamic dual-network structure formed by Schiff-base linkages and catechol–Fe^3+^ coordination endowed the dressing with excellent toughness, conductivity, adhesion, and self-healing capability, enabling it to flexibly adapt to skin deformation during wound healing.

## 5. Translational Perspective: Market Landscape, Patents, and Challenges

### 5.1. Market Landscape and Commercialization Drivers

With the advancement of regenerative medicine and advanced wound management technologies, the market potential of polysaccharide-based hydrogels in skin regeneration has become increasingly evident.

The global wound dressing and skin regeneration market continues to expand, driven by population aging, the rising prevalence of diabetes, and the growing number of patients suffering from chronic wounds.

Owing to their excellent biocompatibility, biodegradability, and tunable functionality, natural polysaccharide hydrogels offer distinct advantages in hemostasis, antimicrobial activity, inflammation modulation, and tissue repair, making them a major focus of commercial research and development.

In addition, emerging delivery modalities—such as 3D printing, sprayable and implantable hydrogels, and smart responsive systems—provide polysaccharide hydrogel products with differentiated competitive advantages.

Key industrial drivers include high–value-added functional modifications, controllable drug delivery performance, compatibility with existing medical devices, and safety and reproducibility that meet regulatory requirements.

Together, these factors are accelerating the clinical translation and market adoption of polysaccharide-based hydrogels.

### 5.2. Representative Patents of Polysaccharide-Based Hydrogels for Skin Regeneration

In recent years, the number of patent applications related to polysaccharide hydrogels for skin regeneration has increased markedly, covering multiple aspects such as functional design, composite structures, smart responsiveness, and drug-loading capability [187]. Representative patents demonstrate that the introduction of antibacterial, antioxidant, immunomodulatory, and pro-angiogenic modules enables multi-target synergistic effects in wound repair [188]. Meanwhile, innovative designs—including dynamic crosslinking, injectable hydrogels, and thermoresponsive or ROS-responsive hydrogels—have significantly improved product applicability and clinical handling convenience. Representative patents are summarized in Table 3, highlighting the key technologies, innovations, and applications of polysaccharide-based hydrogels for skin regeneration.

These patents not only reflect technological advances in fundamental material modification but also highlight the strong potential of polysaccharide hydrogels for translation into commercially viable products.

### 5.3. Current Challenges and Translational Barriers

Despite the significant advantages of natural polysaccharide hydrogels in skin regeneration, their commercialization and clinical translation still face multiple challenges.

First, achieving precisely controllable mechanical properties, long-term stability, and batch-to-batch consistency remains difficult under large-scale industrial production conditions.

Second, the long-term safety and efficacy of drug-loaded or functionally modified hydrogels require further comprehensive in vitro and in vivo validation, particularly for applications involving chronic wounds and immune response modulation.

Moreover, the performance controllability and storage stability of smart responsive hydrogels under complex wound microenvironments still need substantial optimization.

In addition, clinical translation requires multidisciplinary collaboration across materials science, pharmaceutics, tissue engineering, and medical device regulation.

From a regulatory perspective, polysaccharide-based hydrogels must comply with medical device biocompatibility standards such as ISO 10993 [189], and long-term in vivo safety data—particularly regarding the metabolism of degradation products of smart responsive hydrogels—must be validated through multicenter clinical trials.

These requirements inevitably increase approval timelines and costs, representing major barriers to current translation efforts.

To address these challenges, future research should focus on material standardization, safety evaluation of functional modules, optimization of controlled release mechanisms, and the development of user-friendly designs compatible with existing clinical procedures, thereby facilitating the industrialization and clinical implementation of natural polysaccharide hydrogels in skin regeneration.

## 6. Conclusions

Natural polysaccharide-based hydrogels, owing to their excellent biocompatibility, biodegradability, multifunctionality, and structural tunability, have emerged as an important class of biomaterials for skin wound repair and tissue regeneration.

This review systematically summarizes recent advances in material types, crosslinking strategies, mechanisms of action, and application studies of polysaccharide hydrogels, clarifying their multidimensional roles in promoting skin regeneration.

From a tissue engineering perspective, natural polysaccharide hydrogels not only function as physical barriers and moist wound dressings, but also mimic the extracellular matrix through three-dimensional porous architectures, thereby supporting cell adhesion, migration, and proliferation, and promoting angiogenesis and tissue remodeling. As a result, these hydrogels exhibit favorable biomimetic properties and bio-instructive functions.

Meanwhile, through the integration of functional modules (such as antibacterial, antioxidant, immunomodulatory, and pro-angiogenic components) and smart responsive designs (including pH-, ROS-, and glucose-responsive systems), polysaccharide hydrogels enable dynamic regulation of the wound microenvironment and multi-target intervention.

Consequently, they demonstrate synergistic therapeutic advantages in coordinating inflammation control, infection prevention, and tissue repair during both acute and chronic wound healing.

In addition, advanced material design strategies—such as double-network architectures, gradient structures, and dynamic crosslinking—have significantly enhanced the mechanical properties, self-healing capability, and injectability of hydrogels, allowing them to better adapt to complex wound geometries and dynamic healing processes.

Despite the promising therapeutic efficacy demonstrated by natural polysaccharide hydrogels in fundamental studies and animal models, their clinical translation still faces substantial challenges.

First, variations in the sources and molecular structures of natural polysaccharides may lead to batch-to-batch performance fluctuations, hindering large-scale production and standardized application.

Second, the long-term biocompatibility, degradation behavior, and in vivo metabolic pathways of complex functional components—such as nanoparticles, growth factors, and exosomes—require systematic evaluation.

Moreover, in real wound environments, interindividual heterogeneity and dynamic changes in microenvironmental signals may compromise the precision and reliability of smart responsive systems.

Finally, most current studies remain largely descriptive, and the specific synergistic mechanisms of polysaccharide hydrogels within multi-signaling pathways, cell–material interactions, and the microbiota–immune–repair network warrant deeper investigation.

To facilitate the clinical translation and industrial development of natural polysaccharide hydrogels, future research should focus on several key directions.

First, standardized material systems and modular functional platforms should be established by unifying extraction, modification, and characterization protocols of natural polysaccharides, and by developing modular functional unit libraries to enable “plug-and-play” hydrogel construction.

Second, by integrating microfluidics, 3D printing, and bioink technologies, intelligent and personalized therapeutic platforms can be developed to fabricate patient-specific hydrogel dressings with tailored wound conformity and individualized therapeutic dosing.

Third, advanced tools such as multi-omics approaches, organoid models, and in situ imaging should be employed to elucidate the molecular and cellular mechanisms of hydrogel-mediated wound repair, while establishing comprehensive evaluation systems covering biosafety, functional durability, and immune responses.

Finally, strengthened collaboration with medical device regulatory agencies is essential to promote the development of GMP-compliant manufacturing processes and to conduct multicenter clinical trials, thereby validating the therapeutic efficacy of polysaccharide hydrogels in complex wounds such as diabetic foot ulcers, burns, and chronic ulcers.

In conclusion, natural polysaccharide-based hydrogels are evolving from “passive dressings” toward “actively regulated tissue engineering platforms.”

Through continuous material innovation, mechanistic elucidation, and translational research, they are expected to become a core component of next-generation intelligent skin regeneration systems, providing robust support for precise, efficient, and personalized wound therapy.

## Figures and Tables

**Table 2:** Representative patents on natural polysaccharide-based hydrogel systems for wound healing applications.

Function	Material	Design Strategy	Mechanism	Application Example	Key References
Rapid Hemostasis	Chitosan, Sodium alginate	Dynamic crosslinking (Schiff base bonds, ionic crosslinking), nanoparticle reinforcement	Aggregation of red blood cells and platelets; activation of coagulation pathways	Traumatic wounds, surgical incisions, burn-related bleeding	[128,177]
Antimicrobial	Chitosan derivatives, silver nanoparticles, photothermal agents	Cationic charge interaction, controlled release, photothermal responsiveness	Disruption of bacterial membranes; inhibition of biofilm formation	Acute wounds, chronically infected wounds	[128,133]
Immunomodulation	Hyaluronic acid, platelet-rich plasma (PRP)	Immune signaling regulation, macrophage polarization (M1→M2)	Modulation of macrophage phenotype; suppression of excessive inflammation	Accelerated wound healing	[132,154]
Re-epithelialization	Hyaluronic acid, gelatin	Three-dimensional porous architecture, cell-adhesive motifs	Promotion of keratinocyte migration and proliferation	Full-thickness skin wound repair	[178,179]
Angiogenesis	VEGF, DFO, oxidized hyaluronic acid	Sustained release, hypoxia-responsive design	Stimulation of endothelial cell proliferation and neovascularization	Diabetic foot ulcers, chronic wounds	[179]
Anti-oxidative	Hydroxybutyl chitosan (GA-modified)	Sustained release of therapeutic agents, free radical scavenging	Reduction in ROS levels and mitigation of oxidative stress	Pressure ulcers, chronic ulcers	[139,180]
Tissue Engineering Scaffold	GelMA, chitosan/gelatin composites, CMCS	Biomimetic fibrous structure, electrical conductivity, dynamic mechanical tuning	Support of cell adhesion, migration, and differentiation	Artificial skin, functional skin regeneration	[42,136,144]
Self-healing	Schiff base/catechol–Fe^3+^ dual networks	Reversible crosslinking, dynamic network architecture	Restoration of mechanical integrity and adaptation to skin motion	Stretchable wounds, dynamic wound environments	[136,144]

**Table 3:** Summary of patents related to polysaccharide-based hydrogels.

Patent No.	Publication/Grant Year	Country/Region	Main Technology	Key Innovation and Application
WO2020225336A1	2020	International (PCT/WIPO)	Polysaccharide–collagen composite hydrogel scaffold	An interpenetrating network hydrogel composed of polysaccharides and collagen, designed to accelerate wound healing and for wound dressing fabrication.
US20220218868A1	2022	United States	Polysaccharide–collagen hydrogel	U.S. family member of WO2020225336A1, applied in wound treatment and cell culture applications.
AU2020267858B2	2025	Australia	Polysaccharide–collagen hydrogel dressing	A polysaccharide–collagen composite system for wound management and enhancement of cellular activity.
US11254754B2	2022	United States	Rapid-gelling biocompatible hydrogel	Photo-initiated crosslinking enables rapid gelation; suitable for drug loading to promote wound healing.
US20210244846A1	2021	United States	Anti-biofilm hydrogel	A functional hydrogel system targeting biofilm-associated infections in chronic wounds.
WO2025011657A1	2025	International (PCT/WIPO)	Multifunctional chronic wound dressing	Polysaccharide-based hydrogel integrated with bioactive components for multi-target repair of chronic wounds.
US9700650B2	2017	United States	Polysaccharide-based hydrogel composition	Composite hydrogels combining polysaccharides and hydrophilic polymers for moist wound management.
WO2010132857A1	2010	International (WIPO)	Biodegradable hydrogel skin scaffold	Polysaccharide-based biodegradable hydrogel scaffold for skin regeneration and wound healing.
US8524271B2	2013	United States	Polysaccharide foam/gel dressing	Tremella polysaccharide/alginate composite wound dressing to reduce infection risk.
CN104415049A	2015	China	Wound healing composition	Hyaluronic acid–based wound healing compositions for skin defects and ulcer treatment.
CN111450312B	2022	China	Heparin–bFGF delivery hydrogel dressing	A polysaccharide-based hydrogel incorporating heparin and bFGF for sustained growth factor release and enhanced chronic wound healing.
CN118217445A	2024	China	Preparation and application of composite hydrogels	Gelatin-based composite hydrogel for skin wound repair, highlighting polysaccharide/protein hybrid characteristics.
CN113924132A	2023	China	Polysaccharide/collagen network hydrogel	Polysaccharide-based composite hydrogel and dressing for wound care applications.
CN112316108B	2025	China	Multicomponent immunomodulatory composition	A hydrogel composition containing polysaccharides (e.g., chitosan, alginate, hyaluronic acid) combined with immunomodulatory proteins.

## Data Availability

This article is a review and does not report new experimental data. All data referenced in this review are cited from previously published studies, which are available in the cited references.
